# Individual and joint effects of metformin and statins on mortality among patients with high‐risk prostate cancer

**DOI:** 10.1002/cam4.2862

**Published:** 2020-02-08

**Authors:** Xiang‐Lin Tan, Jian‐Yu E, Yong Lin, Timothy R. Rebbeck, Shou‐En Lu, Mingyi Shang, William K. Kelly, Anthony D'Amico, Mark N. Stein, Lanjing Zhang, Thomas L. Jang, Isaac Yi Kim, Kitaw Demissie, Anna Ferrari, Grace Lu‐Yao

**Affiliations:** ^1^ Rutgers Cancer Institute of New Jersey Rutgers, The State University of New Jersey New Brunswick NJ USA; ^2^ Department of Epidemiology School of Public Health Rutgers, The State University of New Jersey Piscataway NJ USA; ^3^ Department of Medicine Robert Wood Johnson Medical School Rutgers, The State University of New Jersey New Brunswick NJ USA; ^4^ Department of Epidemiology Bloomberg School of Public Health The Johns Hopkins University Baltimore MD USA; ^5^ Department of Biostatistics School of Public Health Rutgers, The State University of New Jersey Piscataway NJ USA; ^6^ Dana Farber Cancer Institute Harvard TH Chan School of Public Health Boston MA USA; ^7^ Department of Interventional Radiology School of Medicine Tongren Hospital Shanghai Jiao Tong University Shanghai China; ^8^ Department of Medical Oncology Sidney Kimmel Cancer Center at Jefferson Sidney Kimmel Medical College Philadelphia PA USA; ^9^ Sidney Kimmel Cancer Center at Jefferson Philadelphia PA USA; ^10^ Brigham and Women's Hospital and Dana Farber Cancer Institute Boston MA USA; ^11^ Department of Pathology University Medical Center of Princeton Plainsboro NJ USA; ^12^ Department of Biological Sciences Rutgers, The State University of New Jersey Newark NJ USA; ^13^ Jefferson College of Population Health Philadelphia PA USA

**Keywords:** all‐cause mortality, high‐risk prostate cancer, metformin, population‐based cohort, prostate‐cancer mortality, statins, time‐varying Cox proportional hazard models

## Abstract

**Background:**

Pre‐clinical studies suggest that metformin and statins may delay prostate cancer (PCa) metastases; however, data in humans are limited. To the best of our knowledge, this is the first human study aimed to quantify the individual and joint effects of statin and metformin use among patients with high‐risk PCa.

**Methods:**

This population‐based retrospective cohort study identified patients from the Surveillance, Epidemiology, and End Results (SEER)‐Medicare linked database. Exposure to metformin and statins was ascertained from Medicare Prescription Drug Event files. The association with all‐cause and PCa mortality were evaluated using Cox proportional hazard model with competing causes of death, where propensity scores were used to adjusted imbalances in covariates across groups.

**Results:**

Based on 12 700 patients with high‐risk PCa, statin alone or in combination with metformin was significantly associated with reduced all‐cause mortality (Hazard Ratio [HR]: 0.89; 95% Confidence Interval [CI]: 0.83, 0.96; and HR: 0.75; 95% CI, 0.67‐0.83, respectively) and PCa mortality (HR, 0.80; 95% CI: 0.69, 0.92) and 0.64; 95% CI, d 0.51‐0.81, respectively. The effects were more pronounced in post‐diagnostic users: combination use of metformin/statins was associated with a 32% reduction in all‐cause mortality (95% CI, 0.57‐0.80), and 54% reduction in PCa mortality (95% CI, 0.30‐0.69). No significant association of metformin alone was observed with either all‐cause mortality or PCa mortality.

**Conclusions:**

Statin use alone or in combination with metformin was associated with lower all‐cause and PCa mortality among high‐risk patients, particularly in post‐diagnostic settings; further studies are warranted.

## Graphical abstract

In this population‐based cohort study of 12 700 high‐risk prostate cancer patients, statin use alone or combined with metformin was significantly associated with lower all‐cause mortality, particularly in post‐diagnostic settings. Randomized clinical trials are warranted to confirm these findings.

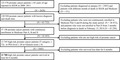

## 1 INTRODUCTION

Approximately 15% of patients diagnosed with prostate cancer (PCa) patients have high‐risk PCa as defined by ≥T2c, or PSA ≥20, or Gleason Score ≥8.^1^ Patients with high‐risk PCa have a significant chance of developing systemic or local recurrence and are at higher risk of death from the disease. Therefore, thus, tremendous attempts to further reduce PCa mortality are directed at these patients. The majority of patients with PCa who later develop lethal metastatic disease have high‐risk localized disease at presentation, also emphasizing the importance of effective treatment strategies at this stage.^2^ Identifying or developing additional therapies with low toxicity and cost is important to improve longevity and quality of life of men diagnosed with high‐risk PCa.

Evidence suggests that two widely prescribed drugs with established safety profiles, metformin and statins, have promising anti‐cancer effects and are associated with lower mortality in PCa patients.^3^, ^4^ Several epidemiologic studies have investigated the effects of individual use of metformin and statins on PCa incidence and mortality.^5^, ^6^, ^7^, ^8^, ^9^ Recently, pre‐clinical data indicated a combination of metformin and statin was better than either drug alone in inhibiting primary tumor growth, metastasis to bone, and biochemical failure; human data on the joint effects of statins and metformin on PCa mortality are limited.^10^, ^11^ Because the majority of metformin users also take statins^12^, ^13^ some of the favorable outcomes observed among metformin users might be derived from statins.

It is essential to distinguish the individual and joint effects of statins and metformin to further understand their potential synergistic role in cancer, explore alternative therapeutics for preventing PCa progression, and to inform future trial design. The primary objective of this study was to quantify the individual and joint effects of metformin and statins on all‐cause and PCa mortality, and to test the hypothesis that a combination of metformin and statin is associated with lower all‐cause and PCa mortality among high‐risk patients. In order to understand the role of statins or metformin in an adjuvant setting, we specifically sought to quantify the individual and joint effects of statins and metformin in post‐diagnostic settings (ie, medications initiated after PCa diagnosis).

## 2 MATERIALS AND METHODS

### 2.1 Data sources and study participants

We used data from the Surveillance, Epidemiology, and End Results (SEER‐18) database linked with Medicare files. The SEER program covers about 28% of the US population, collects information on newly diagnosed cancer patients living in predefined US geographical areas, with about 98% ascertainment rate.^14^

**Figure 1:**
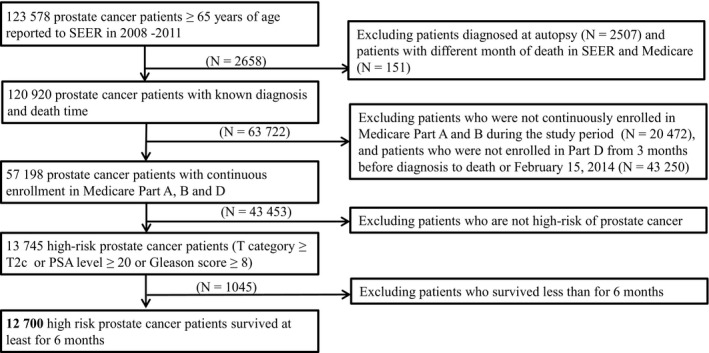
Selection of patients diagnosed with primary high‐risk prostate cancer in SEER 2008‐2011

**Figure 2:**
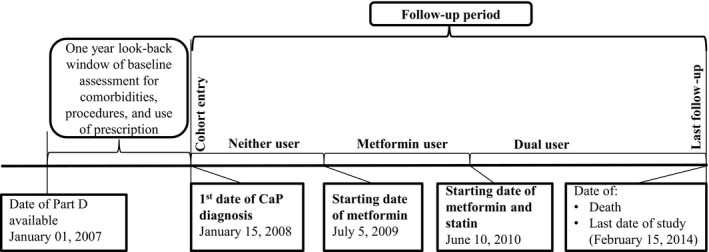
Timeline of a patient's baseline status and exposure through the study. The follow‐up starts on the date of prostate cancer diagnosis, January 15, 2008 (cohort entry date). The 1‐yr look‐back period for comorbidities, procedures, and use of prescription started on January 1, 2007. The end of follow‐up date is February 15, 2014

Our study includes patients diagnosed with cancer through 2011, and Part D data 2007‐2012. To allow a 1‐year window before or after cancer diagnosis for baseline assessment of comorbidities and use of prescriptions, we selected patients with high‐risk PCa being diagnosed from January 2008 to December 2011 (Figures 1 and 2).

Primary PCa cases were identified using the *International Classification of Disease for Oncology, Third Edition* (ICD‐O‐3) histology codes (https://seer.cancer.gov/icd-o-3/). Patients were excluded if enrolled in health‐care maintenance organizations, diagnosed at autopsy, missing a diagnosis date, or had a death date equal to or less than the diagnosis date. To ensure that Medicare claims were available for all participants, we included patients who were continuously enrolled in Medicare Part A and B during the study period (January 2007 to February 2014). To capture the potential drug effect up to 3 months before cancer diagnosis, we further restricted our study to those who were continuously enrolled in Medicare Part D beginning at least 3 months prior cancer diagnosis until death or December 31, 2012, the last date of available Part D data. All patients were assigned into low‐, intermediate‐, or high‐risk groups based on D'Amico classification system.^15^ We selectively focused on high‐risk PCa (T category ≥T2c or prostate specific antigen level ≥20 or Gleason score ≥8; equivalent to overall cancer stage ≥IIB), because evidence regarding statin effects are most consistent for advanced PCa.^3^ Moreover, because the follow‐up time of this study was relatively short, precise estimates of PCa mortality endpoints could be reached in high‐risk patients only. To account for latency effects and minimize healthy user effect (statins are usually discontinued in individuals with short life expectancy), we further restricted our study to those who survived at least 6 months. The study was conducted in accordance with a SEER‐Medicare data use agreement and approved by the Institutional Review Board at Rutgers University.

### 2.2 Metformin and statin exposures

To determine metformin and statin exposures, Part D prescription drug event files were used. These files include information on drug name, dispensation dates, dosage, and days’ supply for each prescription. We defined post‐diagnostic users as patients who had their first documented prescription of study medication after the PCa diagnosis. Patients who had first documented prescription of study medication before the PCa diagnosis and continuously used the drug after diagnosis were considered pre‐diagnostic users. Statins were categorized based on the following drug characteristics: lipophilic (atorvastatin, fluvastatin, lovastatin, and simvastatin); hydrophilic (pravastatin and rosuvastatin); high potency (atorvastatin, rosuvastatin, and simvastatin); or low potency (fluvastatin, lovastatin, and pravastatin).^16^

### 2.3 Assessment of covariates and study outcomes

Information was extracted on age, race, marital status, region, year of diagnosis, state buy‐in (for individual with limited income and resources, the state pays part or all of the patient's Medicare Part B premium or the person is in the Medicaid program), as well as contextual data on socioeconomic status including income and education. To avoid overestimating comorbidity as a result of PCa diagnosis, the Charlson score was based on diagnosis codes from 11 months prior to cancer diagnosis.^17^ Receipt of primary cancer therapy was ascertained within 1 year of cancer diagnosis,^18^ and the presence of secondary cancer therapy was ascertained if patients switched to other cancer therapies after a period of primary therapy.

The primary endpoints were all‐cause and PCa mortality. Data on all‐cause and PCa mortality were based on SEER records available through February 15, 2014 and December 15, 2011, respectively (Figure 2). Causes of PCa death in the SEER record were based on the underlying causes of death in the death certificate, which had a high agreement (87%‐92%) with medical record review.^19^ Patients were censored at death, last contact, or the last date of available data for endpoints.

### 2.4 Statistical analyses

Descriptive statistics with Chi‐square tests were used to estimate the differences on demographic and clinical characteristics. Propensity scores 20 were used to estimate the probability of one of the following four exclusive medication use categories: “ever used statin” (p1), “ever used metformin” (p2), “ever used both statin and metformin” (p3) and “none.” They were calculated based on patients’ sociodemographic characteristics, and comorbidities (Charlson Comorbidity Score, diabetes, obesity/metabolic syndrome, and hyperlipidemia) using a general (polytomous) logistic regression model.^21^ To control pre‐treatment imbalances on observed variables, propensity scores p1, p2, and p3 were included as covariates in the statistical models for propensity score adjustment. Cox proportional hazards models (with competing causes of death) were used to estimate hazards ratios (HRs) and 95% confidence intervals (CIs).^22^, ^23^

The association between metformin and/or statin use and all‐cause and PCa mortality was evaluated by sequentially adding the following variables: (a) demographic characteristics; (b) tumor characteristics; (c) treatment characteristics; (d) comorbidities, and (e) Charlson Comorbidity Score. We developed the final model by adjusting for cancer stage, primary cancer therapy (surgery, radiotherapy, androgen deprivation therapy [ADT]), secondary cancer therapy, salvage radiation, and propensity scores, as well as the variables that remained imbalanced after propensity score adjustment (ie, age, Charlson Score, diabetes, dyslipidemia, diabetes/IGT). Cancer stage were grouped based on five key components (T category, N category, M category, PSA level and Gleason core) using the American Joint Committee on Cancer TNM staging system. We included overall cancer stage instead of individual component to reduce a large number of variables in Cox models. For all of the models we considered, the proportional hazards assumption was evaluated using Schoenfeld residual plots.^24^ All analyses were two‐tailed test based on α = 0.05 and conducted using SAS Version 9.4 (SAS Institute).

### 2.5 Sensitivity and subgroup analyses

According to the new National Comprehensive Cancer Network risk criteria,^25^ patients with a clinical stage of T2c were no longer considered to have high‐risk PCa. Therefore, we performed a sensitivity analysis using the study population including all patients with clinical stage ≥T3a, or Gleason score ≥8, or PSA ≥20 ng/mL. To address potential healthy user effects and latency for analyses of post‐diagnostic statin use, we restricted our study to those who survived at least for 6 months. However, potential bias could result from excluding the sickest cases for analyses of pre‐diagnostic statin use, we further performed a sensitivity analysis by including the patients with survival less than 6 months. Additionally, it is important to know whether the drug effect varies with patient characteristics, other cancer therapies received, or other prognostic factors; therefore, we carried out multiple pre‐planned sub‐analyses by: (a) primary cancer therapy; (b) presence of secondary cancer therapy, suggesting that the patients have failed primary cancer therapy; (c) cancer stage and M categories; (d) statin name, type, and potency; and (e) presence of documented obesity or other major comorbidities.

## 3 RESULTS

### 3.1 Baseline characteristics

The study cohort consisted of 12 700 high‐risk PCa patients who were diagnosed between 2008 and 2011, and survived at least for 6 months. The median age at diagnosis was 74 years (quartiles: 70‐80). During a median follow‐up of 42 months (quartiles: 26.4‐57.6), 2182 (17.2%) patients died from any cause and 1078 (8.5%) died from prostate cancer. Most metformin users took statins (1911/2346 = 81%, Table 1). Compared to those who did not use statins or metformin, users of metformin alone, statin alone, and their combination were significantly younger and more likely to have diabetes, obesity or metabolic syndrome, and hyperlipidemia, and had a higher Charlson score with less advanced‐stage cancer (Table 1).

**Table 1:** Demographic, clinical, and comorbid characteristics

Variables	No. (%) of patients					*P*	Adjusted *P* e
	Overall (n = 12,700)	No metformin/no statin (n = 4568)	Metformin alone (n = 435)	Statin alone (n = 5786)	Metformin+statin (n = 1911)		
Age						<.01	.04
65‐69	3123 (25)	1117 (24)	122 (28)*	1328 (23)**	556 (29)**		
70‐79	6344 (50)	2150 (47)	218 (50)	2938 (51)	1038 (54)		
80+	3233 (25)	1301 (28)	95 (22)	1520 (26)	317 (17)		
Marital status						<.01	.98
No	3307 (26)	1300 (28)	117 (27)	1436 (25)**	454 (24)**		
Yes	7806 (61)	2696 (59)	258 (59)	3653 (63)	1199 (63)		
Unknown	1587 (12)	572 (13)	60 (14)	697 (12)	258 (14)		
Race						<.01	.96
White	9805 (77)	3495 (77)	307 (71)*	4597 (79)**	1406 (74)**		
Black	1652 (13)	678 (15)	77 (18)	619 (11)	278 (15)		
Others	1243 (10)	395 (9)	51 (12)	570 (10)	227 (12)		
Region						<.01	.72
Northeast	1651 (13)	569 (12)	60 (14)	781 (14)*	241 (13)		
South	2625 (21)	970 (21)	113 (26)	1123 (19)	419 (22)		
North central	1146 (9)	445 (10)	37 (9)	501 (9)	163 (9)		
West	7278 (57)	2584 (57)	225 (52)	3381 (58)	1088 (57)		
Education^a^						<.01	.73
>92%	4079 (32)	1451 (34)	102 (23)**	1997 (35)**	529 (28)**		
80%‐92%	4556 (36)	1656 (36)	173 (40)	2025 (35)	702 (37)		
< 80%	3434 (27)	1249 (27)	134 (31)	1457 (25)	594 (32)		
Unknown	631 (5)	212 (5)	26 (6)	307 (5)	86 (5)		
Median income						<.01	.86
≤$45 000	3833 (30)	1459 (32)	155 (36)*	1603 (28)**	616 (32)		
$45000‐$70 000	4238 (33)	1504 (33)	154 (35)	1908 (33)	672 (35)		
>$70 000	4021 (32)	1401 (31)	101 (23)	1980 (34)	539 (28)		
Unknown	608 (5)	204 (4)	25 (6)	295 (5)	84 (4)		
Year of diagnosis						<.01	.97
2008	4028 (32)	1407 (31)	142 (33)	1829 (32)*	650 (34)*		
2009	3734 (29)	1298 (28)	116 (27)	1771 (31)	549 (29)		
2010	2529 (20)	939 (21)	82 (19)	1127 (19)	381 (20)		
2011	2409 (19)	924 (20)	95 (22)	1059 (19)	331 (17)		
State buy‐in^b^						<.01	.54
No	9542 (75)	3407 (75)	300 (69)*	4503 (78)**	1332 (70)**		
Yes	3158 (25)	1161 (25)	135 (31)	1283 (22)	579 (30)		
Charlson score^c^						<.01	.03
0	7970 (63)	3426 (75)	207 (48)**	3568 (62)**	769 (40)**		
1	2004 (16)	567 (12)	114 (26)	926 (16)	397 (21)		
2+	2726 (21)	575 (13)	114 (26)	1292 (22)	745 (39)		
Diabetes						<.01	<.01
No	6928 (55)	3244 (71)	65 (15)**	3375 (58)**	244 (13)**		
Yes	5772 (45)	1324 (29)	370 (85)	2411 (42)	1667 (87)		
Obesity/metabolic syndrome						<.01	.01
No	10 806 (85)	4123 (90)	356 (82)**	4924 (85)**	1403 (73)**		
Yes	1894 (15)	445 (10)	79 (18)	862 (15)	508 (27)		
Hyperlipidemia						<.01	<.01
No	4316 (34)	2653 (58)	187 (43)**	1138 (20)**	338 (18)**		
Yes	8384 (66)	1915 (42)	248 (57)	4648 (80)	1576 (82)		
Cancer stage						<.01	NC
IIB	8546 (67)	2902 (64)	270 (62)	4023 (70)**	1351 (71)**		
III	1259 (10)	457 (10)	53 (12)	559 (10)	190 (10)		
IV	2895 (23)	1209 (26)	112 (26)	1204 (21)	370 (19)		
M categories						<.01	NC
M0	9646 (76)	3281 (72)	309 (71)	4542 (78)**	1513 (79)**		
M1	2412 (19)	1066 (23)	101 (22)	942 (16)	303 (16)		
Unknown	642 (5)	221 (5)	25 (5)	302 (5)	95 (5)		
Lymph notes						<.01	NC
No	10 283 (81)	3598 (79)**	336 (77)**	4773 (82)**	1576 (82)**		
Yes	971 (8)	390 (9)	44 (10)	407 (7)	130 (7)		
Missing	1446 (11)	580 (13)	55 (13)	606 (10)	205 (11)		
Androgen deprivation therapy^d^						<.01	NC
No	8279 (65)	2980 (65)	250 (57)**	3829 (66)	1220 (64)		
Yes	4421 (35)	1588 (35)	185 (43)	1957 (34)	691 (36)		
Radiation therapy^d^						<.01	NC
No	9535 (75)	3581 (78)	316 (73)**	4278 (74)**	1360 (71)**		
Yes	3165 (25)	987 (22)	119 (27)	1508 (26)	551 (29)		
Chemotherapy^d^						.54	NC
No	12 176 (96)	4374 (96)	418 (96)	5561 (96)	1823 (95)		
Yes	524 (4)	194 (4)	17 (4)	225 (4)	88 (5)		
Surgery^d^						<.01	NC
No	10 890 (86)	4000 (88)	389 (89)	4873 (84)**	1628 (85)**		
Yes	1810 (14)	568 (12)	46 (11)	913 (16)	183 (15)		
Salvage radiation						.08	NC
No	12 356 (97)	4458 (98)	419 (96)	5612 (97)	1867 (98)		
Yes	344 (3)	110 (2)	16 (4)	174 (3)	44 (2)		
Secondary therapy						<.01	NC
No	9297 (73)	3457 (76)**	304 (70)**	4211 (73)**	1325 (69)**		
Yes	3403 (27)	1111 (24)	131 (30)	1575 (27)	586 (31)		
Gleason categories						<.01	.17
<8	2293 (18)	788 (17)	57 (13)**	1082 (19)	366 (19)		
8	2322 (18)	743 (16)	82 (19)	1128 (19)	369 (19)		
>8	2042 (16)	687 (15)	74 (17)	943 (16)	338 (18)		
Missing	6043 (48)	2350 (51)	222 (51)	2633 (46)	838 (44)		

Abbreviations: NC, not calculated; PSA, prostate‐specific antigen.

a Reflecting a percentage of high school education.

b Indicating that the state pays part or all of the patient's medicare part B premium or the person is in the Medicaid program.

c 1 year to 1 month before cancer diagnosis.

d Within 1 year of cancer diagnosis.

e Reflects differences between groups after adjusting for propensity score for metformin, statin, and dual users.

* *P* < 0.05, compared to neither users.

** *P* < 0.01, compared to neither users.

### 3.2 Survival outcomes

Median survival was 3.1 years with metformin, 3.6 with statins, and 3.9 with metformin plus statin, vs 3.1 years for those who did not use either drug. Based on Cox models, metformin plus statin (HR, 0.75; 95% CI, 0.67‐0.83) and statin alone (HR, 0.89; 95% CI, 0.83‐0.96) were significantly associated with a lower all‐cause mortality, after adjusting for potential confounders (Table 2). With respect to PCa mortality, metformin plus statin was associated with a 36% risk reduction (95% CI, 0.54‐0.85), followed by statin alone (HR, 0.80; 95% CI, 0.69‐0.92) (Table 2). Metformin alone was relatively rare, and there was no significant association with all‐cause mortality (HR, 0.89; 95% CI, 0.75‐1.05) (Table 2), and PCa mortality (HR, 0.75; 95% CI, 0.53‐1.05) (Table 2).

**Table 2:** Hazard ratios (HRs) of all‐causes and PCa mortality for metformin and/or statin use in the whole population or pre‐ and post‐diagnostic users

Category	All‐cause mortality		PCa mortality	
	Crude HR (95% CI)	Adjusted HR (95% CI)^a^	Crude HR (95% CI)	Adjusted HR (95% CI)^a^
All study population				
No metformin/no statin	1.0	1.0	1.0	1.0
Metformin alone	1.00 (0.85‐1.17)	0.89 (0.75‐1.05)	0.71 (0.51‐1.00)	0.75 (0.53‐1.05)
Statin alone	0.81 (0.75‐0.86)**	0.89 (0.83‐0.96)**	0.63 (0.55‐0.72)**	0.80 (0.69‐0.92)**
Metformin+statin	0.69 (0.63‐0.76)**	0.75 (0.67‐0.83)**	0.45 (0.36‐0.55)**	0.64 (0.51‐0.81)**
Pre‐diagnostic users				
No metformin/no statin	1.0	1.0	1.0	1.0
Metformin alone	1.08 (0.89‐1.31)	0.91 (0.75‐1.11)	0.75 (0.50‐1.11)	0.76 (0.50‐1.13)
Statin alone	0.87 (0.82‐0.94)**	0.94 (0.87‐1.02)	0.69 (0.61‐0.79)**	0.84 (0.73‐0.98)*
Metformin+statin	0.77 (0.69‐0.86)**	0.82 (0.72‐0.92)**	0.52 (0.41‐0.66)**	0.73 (0.56‐0.95)**
Post‐diagnostic users				
No metformin/no statin	1.0	1.0	1.0	1.0
Metformin alone	0.85 (0.64‐1.13)	0.89 (0.67‐1.19)	0.65 (0.36‐1.18)	0.73 (0.39‐1.35)
Statin alone	0.57 (0.50‐0.64)**	0.73 (0.64‐0.84)**	0.38 (0.28‐0.51)**	0.58 (0.43‐0.78)**
Metformin+statin	0.56 (0.48‐0.66)**	0.68 (0.57‐0.80)**	0.31 (0.21‐0.47)**	0.46 (0.30‐0.69)**

a Adjusted for cancer stage, ADT, radiation therapy, surgery, salvage radiation, secondary cancer therapy, propensity scores, and imbalanced variables after propensity scores adjustment.

* *P* < .05, compared to controls.

** *P* < .01, compared to controls.

To provide insight about the potential impact in an adjuvant setting, we examined the differential effects among pre‐diagnostic and post‐diagnostic users. The effects of statin alone or combination of metformin and statin on both all‐cause and PCa mortality are more pronounced in post‐diagnostic users. Among post‐diagnostic users, statin alone was significantly associated with a 27% reduction in all‐cause mortality (95% CI, 0.64‐0.84) and a 42% reduction in PCa mortality, compared to those who did not use statins or metformin. Metformin plus statins was associated with a 32% reduction in all‐cause mortality (95% CI, 0.57‐0.80), and a 54% reduction in PCa mortality (95% CI, 0.30‐0.69) (Table 2). Again, metformin alone did not show any significant effects on all‐cause mortality and PCa mortality among either pre‐diagnostic or post‐diagnostic users (Table 2).

### 3.3 Sensitivity and subgroup analyses

We first performed sensitivity analyses by using the study population including all patients with clinical stage ≥T3a, or Gleason score ≥8, or PSA ≥20 ng/mL. Similar HRs of all‐causes and PCa mortality were observed for metformin and/or statin use (data not shown). Therefore all of the following subgroup analyses were performed using the study population that included all patients with clinical stage ≥T2c, or Gleason score ≥8, or PSA ≥20 ng/mL. We also performed sensitivity analyses by including the patients with survival less than 6 months, and a similar pattern of the results with even lower HRs were observed (data not shown).

To assess the impact of disease extent on observed drug effects, we stratified medication use by disease extent (ie, cancer stage, M categories), and found no significant effect modification for both all‐cause and PCa mortality (*P_interaction_* > .05) (Table S1). Among patients with Stage IV PCa, data suggest there might be some synergistic effect between statin and metformin (HR, 0.93 for metformin alone, 0.82 for statin alone, and 0.66 for metformin plus statin) although this interaction did not reach statistical significance (*P_interaction_* = .18) (Table S1).

To assess the impact of existing health conditions on observed drug effects, we carried out pre‐planned sub‐analyses by the status of diabetes, dyslipidemia, or obesity/metabolic syndrome. We found that diabetes and dyslipidemia significantly modified the effects of statin alone or metformin plus statin on all‐cause mortality (*P_interaction_* < .0001), but not on PCa mortality (*P_interaction_* ≥ .05) (Table S2). No significant effect modification of obesity/metabolic syndrome was observed for both all‐cause and PCa mortality (*P_interaction_* > .05) (Table S2). To evaluate the effect modification of cancer treatment, we stratified by the status of primary and secondary cancer therapy as well as salvage radiation, we observed no evidence that they modified the effects of metformin and statins (*P_interaction_* > .05) (Tables S3 and S4). To account for the healthy user effects, we further stratified by Charlson score, and found no significant effect modification for both all‐cause and PCa mortality (*P_interaction_* > .05) (Table S5).

Further pre‐planned sub‐analyses revealed that among statins, only lovastatin was not significantly associated with the reduction in PCa mortality (HR, 0.92; 95% CI, 0.76‐1.12) (Table 3). The effects of lipophilic vs hydrophilic and high vs low potency statins were not statistically different (Table 3).

**Table 3:** Hazard ratios for the association between statin use (name, type, and potency) and PCa mortality among post‐diagnostic users

Category	No. of statin users vs Non‐users	Crude HR (95% CI)	Adjusted HR (95% CI)^a^
Brand name^b^, ^c^			
Atorvastatin	350/5003	0.61 (0.52‐0.73)**	0.76 (0.63‐0.91)**
Lovastatin	78/5003	0.96 (0.79‐1.15)	0.92 (0.76‐1.12)
Pravastatin	282/5003	0.53 (0.41‐0.69)**	0.68 (0.52‐0.89)**
Rosuvastatin	120/5003	0.51 (0.36‐0.70)**	0.71 (0.50‐0.99)*
Simvastatin	865/5003	0.73 (0.64‐0.83)**	0.87 (0.75‐0.99)*
Type^b^			
Lipophilic	1165/5003	0.68 (0.60‐0.76)**	0.85 (0.74‐0.97)*
Hydrophilic	385/5003	0.50 (0.40‐0.62)**	0.69 (0.55‐0.85)**
Potency^b^			
Low	353/5003	0.61 (0.54‐0.69)**	0.83 (0.72‐0.95)**
High	1185/5003	0.79 (0.68‐0.93)**	0.86 (0.73‐1.01)

a Adjusted for cancer stage, ADT, radiation therapy, surgery, salvage radiation, secondary cancer therapy, propensity scores, imbalanced variables after propensity scores adjustment, and metformin use.

b Categories are not mutually exclusive for these variables.

c One patient with fluvastatin were excluded from the analysis.

* *P* < .05, compared to non‐statin users.

** *P* < .01, compared to non‐statin users.

## 4 DISCUSSION

Both metformin and statins are individually associated with reduction in PCa mortality and the use of both medications together is common.^3^, ^4^ To the best of our knowledge, this is the first major epidemiological study to quantify individual and joint effects of metformin and statin on all‐cause and PCa mortality among high‐risk PCa patients. We found that both statin alone and a combination of metformin and statin was significantly associated with reduced all‐cause and PCa mortality. The effect of combination use of metformin and statin was particularly substantial among post‐diagnostic users with high‐risk PCa (54% reduction in PCa mortality) despite the relatively short follow‐up time. Based on the existing evidence, a well‐designed clinical trial is warranted to investigate the roles of statins and combination statins/metformin to reduce the mortality of PCa.

Several epidemiological studies have previously investigated the association between statin use and all‐cause or PCa mortality, with encouraging though mixed findings. We observed that use of statin alone or in combination with metformin was significantly associated with reduced all‐cause and PCa mortality. This finding is comparable with the results from several recent publications. For example, a large retrospective cohort study with 249.986 Saskatchewan Men aged ≥40 years reported a substantial protective association between statin use and PCa mortality (HR, 0.73; 95% CI, 0.66‐0.81).^26^ Wu et al reported that compare to non‐users, statin use was associated with significant reductions in all‐cause mortality (HR, 0.75; 95% CI, 0.68‐0.82) and PCa mortality (HR, 0.77; 95% CI, 0.69‐0.86) in locally advanced and metastatic PCa patients.^27^ In addition, using national Veterans Health Administration database, Anderson‐Carter et al identified 87 346 PCa patients on ADT and found that statin use was an independent predictor of improved overall survival (HR, 0.66; 95% CI, 0.63‐0.68) and PCa specific survival (HR, 0.56; 95% CI, 0.53‐0.60).^28^ However, several prior studies did not show an association between this medication and prostate cancer mortality.^29^, ^30^, ^31^ This discrepancy may be due to the different study population across these studies, indicating the findings may apply to clinical heterogeneous of PCa patients. Only two studies aimed to examine the combination effect of metformin and statin on prostate cancer outcomes.^11^, ^32^ One study showed the combination leads to synergistic effects to lower risk of biochemical recurrence after radical prostatectomy,^11^ but the other showed negative results.^32^ To the best of our knowledge, this is the first study distinguishing the individual and joint effects of statins and metformin on all‐cause and PCa mortality in high‐risk PCa patients.

We also found that post‐diagnostic statin use was associated with lower PCa mortality compared to pre‐diagnostic statin use. A recent population‐based cohort study consisting of a general male population of Finland participating in the Finnish Randomized Study for PCa Screening showed that post‐diagnosis statin use but not pre‐diagnostic statin use was significantly associated with a decreased risk of PCa death.^33^ Additionally, Larsen et al reported that post‐diagnostic statin use was associated with a 19% reduction in all‐cause mortality and 17% reduction in PCa mortality among 31 790 Danish PaCA patients. 9 However, these studies did not focus on high‐risk patients and did not adjust for metformin effect. Our finding differs from a published study by Yu et al, which found that decreased risk of PCa mortality was more pronounced when patients used statins both before and after cancer diagnosis.^34^ The discrepancy might stem from differences in study populations and data sources (the study by Yu et al did not include cancer stage, Gleason grade, or PSA in most of the patients).

Our study also revealed that men took atorvastatin, pravastatin, or rosuvastatin, but not lovastatin demonstrated a significant reduction in PCa mortality compared with non‐users, which is consistent with the findings from a recent population‐based cohort study using Taiwan National Health Insurance Research Data.^27^ We have only one patient with fluvastatin and no patient with pitavastatin in our study, therefore no statistic survival analysis is available for the fluvastatin and with pitavastatin. It has been shown that that atorvastatin, pravastatin, and rosuvastatin are more effective at lowering triglycerides and low‐density lipoprotein cholesterol and raising high‐density lipoprotein cholesterol than other statins in patient with hypercholesterolemia.^35^, ^36^ Interestingly, use of atorvastatin was associated with a reduction in PCa mortality (HR, 0.76; 95% CI, 0.63‐0.91), which is consistent with a recent study demonstrating that men on atorvastatin had a longer median time to progression on androgen deprivation therapy compared to non‐users (27.5 vs 17.4 months, *P* = .0005).^37^ The in vitro study further demonstrated that atorvastatin competitively reduced dehydroepiandrosterone sulfate uptake and thus, effectively decreased the available intratumoral androgen pool, affording a plausible mechanism to support the clinical observation.^37^ Although the exact mechanisms remain unknown, it is worth noting that atorvastatin exhibits one of the most potent lipid‐lowering effects per dose of any statin, one of the greatest bioavailability, and one of the longest half‐lives.^38^

Our findings likely apply to most PCa patients because of the broad representation of various racial/ethnic groups in this study. The findings may be limited; however, by the data sources. The SEER‐Medicare database contains limited data on men under age 65. Given that the majority of PCa is diagnosed among patients who are over age 65, our findings likely apply to most PCa patients. It is possible that some of the non‐users might have used statins or metformin prior to 2007, the earliest year of available Part D data. It is worth noting that post‐diagnostic users are much less likely to be affected by prevalent user effect because we have more than one year of claims to verify the use of metformin or statins. Therefore, it is unlikely the misclassification could explain away the large effect observed in post‐diagnostic patients. While immortal time bias might occur among post‐diagnostic users because they need to live long enough to start medications. However, we have used the following approaches to minimize its effects. First, we restricted to the study population to those who survived at least 6 months so that the probability of initiating metformin or statin after PCa diagnosis is about the same across groups. Second, we made adjustment to propensity scores, which estimate the probability of being one of four exclusive treatment groups, to account for the confounding effects associated with various patient characteristics or disease status. Lastly, we aligned their follow‐up time according to time origin for PCa diagnosis so that comparisons are being made across persons at equal distance from time origin. SEER‐Medicare files also lack data on some important confounding variables, including body mass index, smoking, family history of cancer, or use of nonsteroidal anti‐inflammatory drugs, but subgroup and sensitivity analyses showed that our results are relatively robust (Tables S1‐S5).^39^ It is worth noting that dual users of metformin and statin had higher comorbidity scores at the baseline vs non‐users and this might have led to underestimated treatment benefits. Finally, the impact of the drug exposure may not be fully captured and limited sample size of metformin monotherapy users. However, by focusing on high‐risk patients, our study showed clinically meaningful differences in PCa mortality in various sub‐groups.

## 5 CONCLUSION

Our data demonstrated that statin alone is associated with reduced all‐cause and PCa mortality, and combination of metformin and statin holds great promise for reducing all‐cause or PCa mortality among patients with high‐risk PCa, particularly in post‐diagnostic settings. Further sub‐analyses revealed that all brand of statins, except lovastatin, were significantly associated with the reduction in PCa mortality, and the effects of lipophilic vs hydrophilic and high vs low potency statins were not statistically different. The data presented in this paper provide crucial insight for the design of future randomized clinical trials of statin for high‐risk PCa patients.

## CONFLICT OF INTEREST

None declared.

## Data Availability

The data that support the findings of this study are available from the corresponding author upon reasonable request.
