# Supplementary material for: Individual and joint effects of metformin and statins on mortality among patients with high‐risk prostate cancer
Source: Cancer Med. 2020 Feb 8;9(7):2379–89. doi: 10.1002/cam4.2862 (PMC7131852; doi:10.1002/cam4.2862)
Supplement: Supplementary file 1 [file CAM4-9-2379-s001.docx]

**Supplementary Table 1.** HRs of all-causes and PCa mortality for metformin and/or statin use in the whole population or pre- and post- diagnostic users with the NCCN high-risk prostate cancer.

|  | **All-cause Mortality** | | **PCa Mortality** | |
| --- | --- | --- | --- | --- |
| **Category** | **Crude HR**  **(95% CI)** | **Adjusted**  **HR (95% CI)^a^** | **Crude HR**  **(95% CI)** | **Adjusted HR**  **(95% CI)^a^** |
| ***All study population*** | | | | |
| **No metformin/no statin** | 1.00 | 1.00 | 1.00 | 1.00 |
| **Metformin alone** | 1.01 (0.86-1.19) | 0.97 (0.82-1.15) | 0.71 (0.50-0.99)^b^ | 0.74 (0.53-1.04) |
| **Statin alone** | 0.94 (0.88-1.00) | 0.97 (0.91-1.04) | 0.73 (0.64-0.83)^c^ | 0.84 (0.74-0.96)^c^ |
| **Metformin + statin** | 0.86 (0.78-0.96)^c^ | 0.88 (0.80-0.98)^b^ | 0.55 (0.45-0.69)^c^ | 0.68 (0.54-0.84)^c^ |
| ***Pre-diagnostic users*** | | | | |
| **No metformin/no statin** | 1.00 | 1.00 | 1.00 | 1.00 |
| **Metformin alone** | 1.04 (0.85-1.26) | 0.97 (0.79-1.18) | 0.75 (0.50-1.11) | 0.77 (0.53-1.12) |
| **Statin alone** | 0.88 (0.82-0.95)^c^ | 0.93 (0.86-1.00)^b^ | 0.71 (0.62-0.81)^c^ | 0.81 (0.71-0.93)^c^ |
| **Metformin + statin** | 0.77 (0.68-0.86)^c^ | 0.79 (0.70-0.89)^c^ | 0.53 (0.42-0.67)^c^ | 0.64 (0.50-0.82)^c^ |
| ***Post-diagnostic users*** | | | | |
| **No metformin/no statin** | 1.00 | 1.00 | 1.00 | 1.00 |
| **Metformin alone** | 0.81 (0.61-1.08) | 0.85 (0.64-1.12) | 0.51 (0.28-0.93)^b^ | 0.60 (0.31-1.17) |
| **Statin alone** | 0.76 (0.67-0.87)^c^ | 0.86 (0.75-0.98)^b^ | 0.41 (0.31-0.56)^c^ | 0.53 (0.39-0.71)^c^ |
| **Metformin + statin** | 0.92 (0.78-1.09) | 0.98 (0.83-1.17) | 0.43 (0.29-0.66)^c^ | 0.61 (0.40-0.92)^b^ |

^a^ Adjusted for age, race, married status, region, median income, education, state buy-in, cancer stage, ADT, radiation therapy, surgery, salvage radiation, secondary cancer therapy, and Charlson score.

^b^ *P* < .05, compared to controls.

^c^ *P* < .01, compared to controls.

**Supplementary Table 2.** Adjusted HRs of all-cause and PCa mortality for metformin and/or statin use among post-diagnostic users, stratified by the status of diabetes, dyslipidemia, and obesity/metabolic syndrome

| Category |  | Adjusted HR (95% CI)^a^ | | | | | |  | |
| --- | --- | --- | --- | --- | --- | --- | --- | --- | --- |
|  |  | **Diabetes** | | | | **Dyslipidemia** | | **Obesity/Metabolic Syndrome** | |
|  |  | **Yes (N = 2410)** | | **No (N = 4041)** | | **Yes (N = 3333)** | **No (N = 3118)** | **Yes (N = 791)** | **No (N = 5660)** |
| *All-cause Mortality* | | | | | | | | | |
| No metformin/no statin | | | 1.00 | | 1.00 | 1.00 | 1.00 | 1.00 | 1.00 |
| Metformin alone | | | 1.09 (0.90-1.31) | | 0.70 (0.42-1.17) | 1.07 (0.85-1.36) | 1.00 (0.82-1.23) | 1.19 (0.79-1.81) | 0.95 (0.80-1.14) |
| Statin alone | | | 0.94 (0.84-1.06) | | 1.00 (0.91-1.09) | 1.18 (0.80-1.30) | 1.13 (0.98-1.30) | 0.80 (0.64-1.01) | 0.99 (0.93-1.07) |
| Metformin + statin | | | 0.96 (0.84-1.08) | | 0.46 (0.32-0.67)^c^ | 1.11 (0.98-1.26) | 0.95 (0.78-1.17) | 0.89 (0.69-1.15) | 0.91 (0.81-1.04) |
| *PCa Mortality* | | | | | | | | | |
| No metformin/no statin | | | 1.00 | | 1.00 | 1.00 | 1.00 | 1.00 | 1.00 |
| Metformin alone | | | 1.00 (0.57-1.74) | | 0.40 (0.05-3.02) | 0.76 (0.39-1.49) | 1.09 (0.50-2.36) | 0.51 (0.11-2.47) | 0.95 (0. 56-1.60) |
| Statin alone | | | 0.65 (0.44-0.98)^b^ | | 0.61 (0.43-0.87)^c^ | 0.63 (0.44-0.90)^b^ | 0.84 (0.56-1.26) | 0.38 (0.16-0.94)^b^ | 0.66 (0.50-0.86)^c^ |
| Metformin + statin | | | 0.59 (0.37-0.93)^b^ | | 0.44 (0.15-1.35) | 0.58 (0.35-0.96)^b^ | 0.59 (0.26-1.32) | 0.32 (0.12-0.89)^b^ | 0.56 (0.36-0.88)^c^ |

^a^ Adjusted for age, race, married status, region, median income, education, state buy-in, cancer stage, ADT, radiation therapy, surgery, salvage radiation, secondary cancer therapy, and Charlson score.

^b^ *P* < .05, compared to controls.

^c^ *P* < .01, compared to controls.

**Supplementary Table 3.** Adjusted HRs of all-cause and PCa mortality for metformin and/or statin use among post-diagnostic users, stratified by the status of primary cancer therapy.

| Category |  | Adjusted HR (95% CI) | | | | | | | |
| --- | --- | --- | --- | --- | --- | --- | --- | --- | --- |
|  |  | **Androgen deprivation therapy^a^** | | **Radiation Therapy^b^** | | | **No Chemotherapy^c^**  **(N = 6198)** | | **No Surgery^d^**  **(N = 5578)** |
|  |  | **Yes (N = 2231)** | **No (N = 4220)** | **Yes (N = 1510)** | | **No (N = 4941)** |  |  |  |
| *All-cause Mortality* | | | | | | | | | |
| No metformin/no statin | | 1.00 | 1.00 | | 1.00 | 1.00 | 1.00 | 1.00 | |
| Metformin alone | | 0.98 (0.77-1.25) | 0.97 (0.78-1.21) | | 1.16 (0.80-1.69) | 0.94 (0.79-1.13) | 0.98 (0.83-1.16) | 0.98 (0.83-1.15) | |
| Statin alone | | 0. 97 (0.87-1.08) | 0.97 (0.88-1.06) | | 1.01 (0.85-1.20) | 0.96 (0.90-1.04) | 0.97 (0.91-1.05) | 0.98 (0.91-1.05) | |
| Metformin + statin | | 0.89 (0.76-1.04) | 0.90 (0.79-1.03) | | 0.88 (0.69-1.12) | 0.91 (0.81-1.01) | 0.89 (0.80-0.99)^e^ | 0.89 (0.81-0.99)^e^ | |
| *PCa Mortality* | | | | | | | | | |
| No metformin/no statin | | 1.00 | 1.00 | | 1.00 | 1.00 | 1.00 | 1.00 | |
| Metformin alone | | 0.87 (0.45-1.67) | 0.91 (0.41-1.99) | | 1.42 (0.52-3.86) | 0.83 (0.47-1.47) | 0.93 (0.55-1.56) | 0.88 (0.53-1.45) | |
| Statin alone | | 0.74 (0.50-1.09) | 0.53 (0.37-0.76)^e^ | | 0.38 (0.17-0.87)^e^ | 0.65 (0.49-0.86)^f^ | 0.59 (0.44-0.78)^f^ | 0.61 (0.47-0.79)^f^ | |
| Metformin + statin | | 0.54 (0.30-0.97)^e^ | 0.50 (0.28-0.90)^e^ | | 0.21 (0.05-0.92)^e^ | 0.60 (0.39-0.92)^e^ | 0.55 (0.36-0.84)^f^ | 0.52 (0.35-0.79)^f^ | |

^a^ Adjusted for age, race, married status, region, median income, education, state buy-in, cancer stage, radiation therapy, surgery, salvage radiation, secondary cancer therapy, and Charlson score.

^b^ Adjusted for age, race, married status, region, median income, education, state buy-in, cancer stage, ADT, surgery, salvage radiation, secondary cancer therapy, and Charlson score.

^c^ Adjusted for age, race, married status, region, median income, education, state buy-in, cancer stage, ADT, radiation therapy, surgery, salvage radiation, secondary cancer therapy, and Charlson score. Data not shown for patients with chemotherapy due to the limited sample size (N = 253).

^d^ Adjusted for age, race, married status, region, median income, education, state buy-in, cancer stage, ADT, radiation therapy, salvage radiation, secondary cancer therapy, and Charlson score. Data not shown for patients with surgery due to the limited sample size (N = 873).

^e^ *P* < .05, compared to controls.

^f^ *P* < .01, compared to controls.

**Supplementary Table 4.** Adjusted HRs of all-cause and PCa mortality for metformin and/or statin use among post-diagnostic users, stratified by the salvage radiation and secondary cancer therapy.

| Category | Adjusted HR (95% CI) | | | |
| --- | --- | --- | --- | --- |
|  | **No Salvage Radiation^a^**  **(N = 6289)** | | **Secondary Cancer Therapy^b^** | |
|  |  |  | **Yes (N = 1510)** | **No (N = 4941)** |
| *All-cause Mortality* | | | | |
| No metformin/no statin | |  | 1.0 | 1.0 |
| Metformin alone | | 0.98 (0.84-1.16) | 1.27 (0.91-1.78) | 0.92 (0.76-1.11) |
| Statin alone | | 0.97 (0.91-1.04) | 1.05 (0.91-1.22) | 0.95 (0.88-1.03) |
| Metformin + statin | | 0.89 (0.81-0.99)^c^ | 1.00 (0.81-1.22) | 0.87 (0.76-0.98)^c^ |
| *PCa Mortality* | | | | |
| No metformin/no statin | |  | 1.0 | 1.0 |
| Metformin alone | | 0.88 (0.53-1.45) | 1.18 (0.50-2.76) | 0.83 (0.45-1.54) |
| Statin alone | | 0.61 (0.47-0.79)^d^ | 0.58 (0.32-1.04) | 0.62 (0.46-0.83)^d^ |
| Metformin + statin | | 0.52 (0.42-0.68)^d^ | 0.31 (0.11-0.85)^c^ | 0.60 (0.38-0.94)^c^ |

^a^ Adjusted for age, race, married status, region, median income, education, state buy-in, cancer stage, ADT, radiation therapy, surgery, secondary cancer therapy, and Charlson score. Data not shown for patients with salvage radiation due to the limited sample size (N = 162).

^b^ Adjusted for age, race, married status, region, median income, education, state buy-in, cancer stage, ADT, radiation therapy, surgery, salvage radiation, and Charlson score.

^c^ *P* < .05, compared to controls.

^d^ *P* < .01, compared to controls.

**Supplementary Table 5.** Adjusted HRs of all-cause and PCa mortality for metformin and/or statin use among post-diagnostic users, stratified by Charlson Score

| Category | Adjusted HR (95% CI)^a^ | | |
| --- | --- | --- | --- |
|  | **Charlson Score = 0**  **(N = 4576)** | **Charlson Score = 1**  **(N = 922)** | **Charlson Score ≥ 2**  **(N = 953)** |
| *All-cause Mortality* | | | |
| No metformin/no statin | 1.0 | 1.0 | 1.0 |
| Metformin alone | 1.03 (0.80-1.32) | 0.70 (0.49-1.01) | 1.18 (0.90-1.56) |
| Statin alone | 0.94 (0.86-1.02) | 0.98 (0.82-1.17) | 1.02 (0.89-1.18) |
| Metformin + statin | 0.97 (0.83-1.13) | 0.91 (0.71-1.17) | 0.83 (0.70-0.98)^b^ |
| *PCa Mortality* | | | |
| No metformin/no statin | 1.0 | 1.0 | 1.0 |
| Metformin alone | 1.15 (0.57-2.28) | 0.83 (0.27-2.55) | 0.73 (0.29-1.85) |
| Statin alone | 0.57 (0.41-0.79)^c^ | 0.79 (0.37-1.71) | 0.63 (0.35-1.13) |
| Metformin + statin | 0.54 (0.30-0.95)^b^ | 0.65 (0.28-1.52) | 0.40 (0.17-0.94)^b^ |

^a^ Adjusted for age, race, married status, region, median income, education, state buy-in, cancer stage, ADT, radiation therapy, surgery, salvage radiation, and secondary cancer therapy.

^b^ *P* < .05, compared to controls.

^c^ *P* < .01, compared to controls.
